# The Spatiotemporal Expansion of Human Rabies and Its Probable Explanation in Mainland China, 2004-2013

**DOI:** 10.1371/journal.pntd.0003502

**Published:** 2015-02-18

**Authors:** Hong-Wu Yao, Yang Yang, Kun Liu, Xin-Lou Li, Shu-Qing Zuo, Ruo-Xi Sun, Li-Qun Fang, Wu-Chun Cao

**Affiliations:** 1 State Key Laboratory of Pathogen and Biosecurity, Beijing Institute of Microbiology and Epidemiology, Beijing, People’s Republic of China; 2 Department of Biostatistics and Emerging Pathogens Institute, University of Florida, Gainesville, Florida, United States of America; The Global Alliance for Rabies Control, UNITED STATES

## Abstract

**Background:**

Human rabies is a significant public health concern in mainland China. However, the neglect of rabies expansion and scarce analyses of the dynamics have made the spatiotemporal spread pattern of human rabies and its determinants being poorly understood.

**Methods:**

We collected geographic locations and timeline of reported human rabies cases, rabies sequences and socioeconomic variables for the years 2004-2013, and integrated multidisciplinary approaches, including epidemiological characterization, hotspots identification, risk factors analysis and phylogeographic inference, to explore the spread pattern of human rabies in mainland China during the last decade.

**Results:**

The results show that human rabies distribution and hotspots were expanding from southeastern regions to north or west regions, which could be associated with the evolution of the virus, especially the clade I-G. A Panel Poisson Regression analysis reveals that human rabies incidences had significant correlation with the education level, GDP per capita, temperature at one-month lag and canine rabies outbreak at two-month lag.

**Conclusions:**

The reduction in the overall human rabies incidence was accompanied by a westward and northward expansion of the circulating region in mainland China. Higher risk of human rabies was associated with lower level of education and economic status. New clades of rabies, especial Clade I-G, played an important role in recent spread. Our findings provide valuable information for rabies control and prevention in the future.

## Author Summary

Although the number of human rabies cases has slightly decreased since 2008 in mainland China, the rabies seemed to be gradually expanding to the low-incidence or non-epidemic areas. The neglect of rabies expansion and scarce analyses of the dynamics have made the spatiotemporal spread pattern of human rabies and its determinants poorly understood. Here, we integrate multidisciplinary approaches to explore and describe the spread pattern and evolution dynamic of human rabies in mainland China during the last decade. The results indicated that the reduction in the overall human rabies incidence was accompanied by a westward and northward expansion of the circulating region, which could be associated with the evolution of the virus, especially the clade I-G. And the education level, GDP per capita, temperature at one-month lag and canine rabies outbreak at two-month lag were firstly found to be significant correlation human rabies incidences according to the Panel Poisson Regression analysis. Our findings give a relatively complete picture about the human rabies spatiotemporal dynamics and spread pattern, thus provide new insights on risk factors and control strategies for the disease spread.

## Introduction

Rabies is a viral zoonotic infection of the central nervous system caused by a lyssavirus, and its mortality rate is nearly 100% without proper post-exposure prophylaxis (PEP). As one of the most feared diseases throughout human history, rabies is widely distributed throughout the world with high mortality, leading to 55,000 human deaths each year [1]. China has the second highest rate of human rabies in Asia, where domestic dogs are the main source of infection and are the primary vector for human rabies. Towards the end of the last century, China encountered the third wave of human rabies since 1949 [2,3], and the reemerging disease was among the top three causes of human death due to infectious diseases in the country [4]. The rapid increase of domestic dog population and inadequate PEP for humans bitten by dogs were thought to be the important factors driving the high incidence of human rabies in mainland China [5–8]. However, data about the burden of canine rabies in China is limited given the lack of detailed data on the number of domestic dogs and comprehensive rabies surveillance among dogs in the country [9,10].

Although previous studies had revealed the number of human rabies cases slightly decreased since 2008, the rabies seemed to be gradually expanding to the low-incidence or non-epidemic areas due to human-related activities (i.e. human migration, pets keeping) [11,12], which would hinder the goal to eliminate rabies by year 2020 [13]. In order to control the burden of rabies expansion, a comprehensive understanding about the spatiotemporal feature and evolution dynamic of rabies is of great importance. However, the previous studies were limited, giving the hotspots and risk factors for the occurrence of human rabies over the years and the spread dynamic of rabies remain unclear. In this study, we conducted multidisciplinary analyses to characterize the spatiotemporal movement of human rabies cases, to describe the spread pattern and rabies evolution, to identify the risk factors for the occurrence of human rabies cases, which could provide evidence-based guidance for policy-makers and service providers to control and prevent the disease.

## Materials and Methods

### Data collection and management

In China, human rabies is a class B notifiable infectious disease, and information regarding each laboratory-confirmed case must be reported to the Chinese CDC (CCDC) through the National Notifiable Disease Surveillance System (NNDSS) [14]. Data on human rabies cases, including age, gender, occupation and month of onset, from January 2004 to December 2013 in mainland China was obtained from the NNDSS.

Demographic data, gross domestic product (GDP) per capita and education level specific to each county were obtained from the China Bureau of Statistics from the sixth national census in 2010. Average monthly temperature covering 700 surveillance stations in mainland China from 2004 to 2013 were collected from the China Meteorological Data Sharing Service System (http://cdc.cma.gov.cn). Monthly outbreaks of canine rabies at the province level were obtained from official veterinary bulletin from the ministry of agricultural of People’s Republic of China.

For the phylodynamic analysis, the full sequences of the *N* gene of rabies with background information including isolation year, host, and province were retrieved from GenBank and literatures [6,15–19], accessed on April 15, 2014. Then we formed a data set including 219 *N* gene sequences from 19 provinces ranging from 1986 to 2012. According to the results of phylogenetic trees, we chose two main lineages named Clade I and Clade II for the discrete phylogeographic analysis. For all available sequence of the two main lineages, we excluded high homologous sequences with the same background information. Then we formed two datasets for the 141 Clade I and 62 Clade II sequences. The accession numbers and strains’ information used in this study are shown in S1 Table.

### Epidemiological features analysis

The bar chart of monthly incidence was produced to check seasonality, and annual incidence curves were plotted to examine the overall temporal trend. Average annual incidences over the whole study period were compared across gender and age groups, and the proportions of human cases by occupation were calculated. To assess the spatiotemporal distribution of human rabies, map series were created to show the spatial distribution of annual incidence of each county. In addition, to better present the epidemic dynamic of the disease, the number of cases of each province was mapped from 2004 to 2013.

### Spatiotemporal hotspots analysis

Hotspots are important characteristics that can be used to target interventions at most- needed places. The spatial movement of hotspots over time is useful not only in describing the disease spread dynamic but also in assessing the effectiveness of disease control and prevention programs. We evaluated the presence of space-time hotspots using Kulldorff’s spatiotemporal scan statistic implemented in SaTScan software (version 9.0) [20]. In order to find as many as possible spatially refined areas with reasonable LLR values, a discrete Poisson model was fitted to identify space-time hotspots, and 90% of the study period and the areas with 10% of the total population size in mainland China were set as the upper search bounds, respectively. Hotspots were detected using the log likelihood ratio (LLR) test statistic whose significance was evaluated with 999 Monte Carlo samples. Spatiotemporal hotspots were then mapped at the county level, together with a map of diffused counties by year, to show the geographic movement of human rabies after 2004.

### Panel Poisson Regression analysis

To explore potential factors related to spatiotemporal heterogeneity of human rabies, a panel Poisson regression was fitted using STATA software (Version 10.0, StataCorp LP, Texas, USA) for the 2004–2013 period. The monthly number of human rabies for each county was set as the outcome variable, and population number was included as the offset variable. Potential risk factors at the county scale, such as temperature, average education level and GDP per capita, were included as covariates in the analysis. Because the incubation of rabies was average 1 to 3 month and the temperature was of lag effect, we explored the time lags of 0 to 4 months for the number of canine rabies outbreaks and 0 to 2 months for temperature. Univariate analysis was performed to examine the effects of individual variables. Multivariate analysis was performed using the variables with a P-value < 0.05 in the univariate analysis after colinearity among these variables was examined. The percentage change (PC) in incidence in response to the change of a variable by a given amount (5°C for temperature, 1000 yuan for GDP per capita, and one year for education level), was used to determine the impact of each variable on disease incidence. The formula for calculating PC is 100*(exp(coefficient)-1). The 95% confidence interval (CI) and corresponding P-value were estimated. As the data of canine rabies outbreaks were collected only at the province level, we fitted a multivariate model to examine the association between monthly human rabies incidences and monthly numbers of canine rabies outbreaks and monthly temperature at provincial level.

### Evolutionary inference and discrete phylogeographic analysis

Spatiotemporal movement of rabies is often coupled with genetic evolution [19,21]. To explore the evolutionary history of rabies and its association with the spatiotemporal spread, we collected available sequences of the *N* gene of rabies and applied a relaxed-clock Bayesian Markov chain Monte Carlo method [22]. Multiple sequence alignment was performed using Muscle [23] with the default setting. The best-fit nucleotide substitution model for each alignment was carried out by using Akaike information criterion (AIC) implemented in JModeltest2.0.2 [24]. We applied a relaxed-clock Bayesian Markov chain Monte Carlo method to explore the genetic diversity of rabies in the BEAST package v1.8.0 [22]. In order to elucidate phylogeographic spread of Clades I and II in time and space, a Bayesian stochastic search variable selection (BSSVS) approach was used to identify significant transition rates between locations [21]. The spread events between two provinces with a Bayes factor of greater than 3 were examined.

For these analyses, we used an uncorrelated lognormal distribution relaxed molecular clock model [25] along with the GMRF Bayesian Skyride model [26] as a coalescent prior. Two independent runs were undertaken for each analysis. The numbers of MCMC iterations and tree-sampling frequencies were shown in S2 Table. Posterior distributions were inspected to ensure adequate mixing in Tracer v1.5 (http://tree.bio.ed.ac.uk/software/tracer). We used Tree Annotator program in the BEAST package to generate a maximum clade credibility (MCC) tree with a burn-in of 10% of the sampled trees. The MCC trees were visualized using FigTree v1.4.0 (http://tree.bio.ed.ac.uk). To explore the spread events, we used SPREADv1.0.6 [27] to calculate the Bayes factor. The genetic diversity distribution and possible spread events of lineage Clades I and II were mapped at the provincial scale.

## Results

### Epidemiological features of human rabies in mainland China

**Fig 1:**
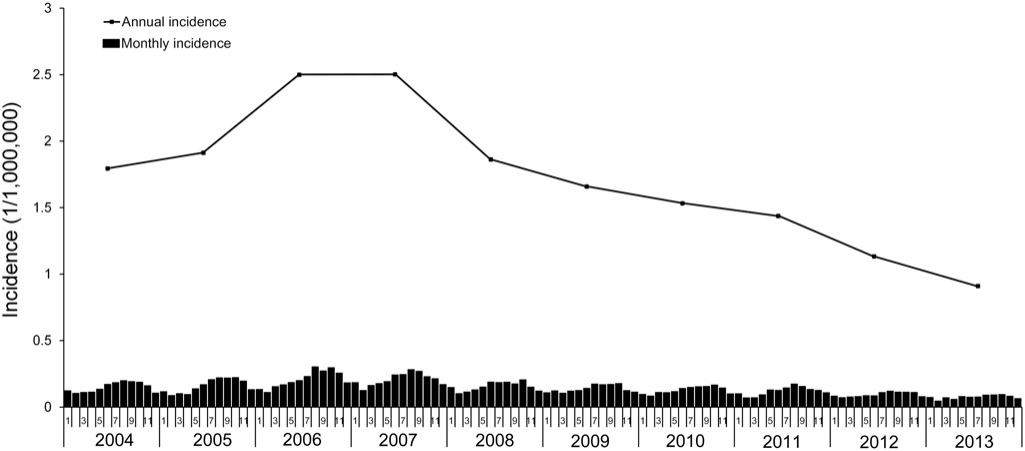
Temporal distribution of human rabies in the mainland China, 2004–2013.

**Fig 2:**
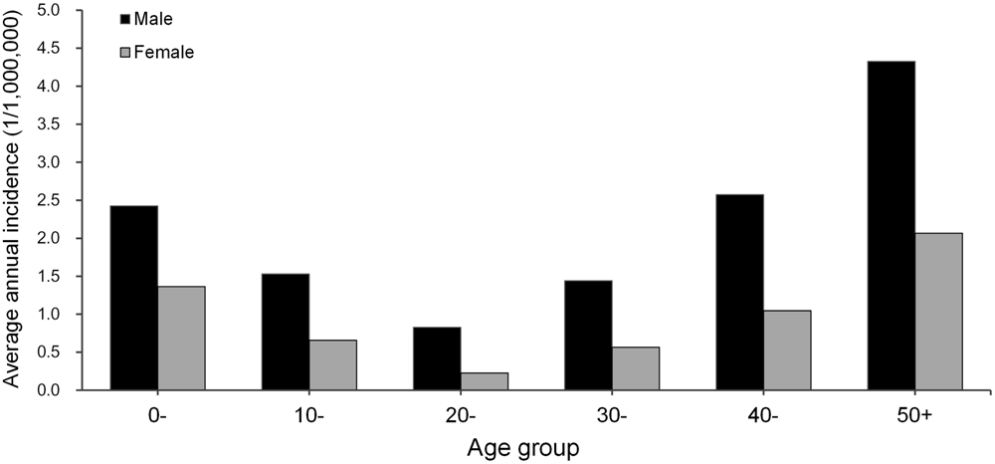
Human rabies incidence over gender and age group in mainland China.

From 2004 to 2013, there were 22,684 cases reported in 30 provinces, across 1821 of 2922 counties in mainland China. The monthly incidence showed a significant seasonal pattern peaking in the Summer and Autumn, especially in the months from August to October each year (Fig. 1). The average seasonal incidence were 5.22 and 5.28 (1/1,000,000) in Summer and Autumn compared 3.62 and 3.32 (1/1,000,000) in Spring and Winter. The annual incidence curve in Fig. 1 showed that the human rabies rapidly increased since 2004, reached its peak in 2006 and 2007 (3267 and 3288 cases), and plunged in 2008 and kept declining slowly afterwards. Males had a significantly higher incidence than females in all age groups (*P* < 0.001), and the total risk ratio was 2.18, and the 50+, 0- and 40- age groups had the highest incidence in both males and females (Fig. 2). In addition, 70.38% of all cases were peasant and herdsman, and followed by student (14.09%) and pre-school children (8.61%). No significantly temporal or spatial heterogeneity was found for the distribution of cases by age or occupation.

**Fig 3:**
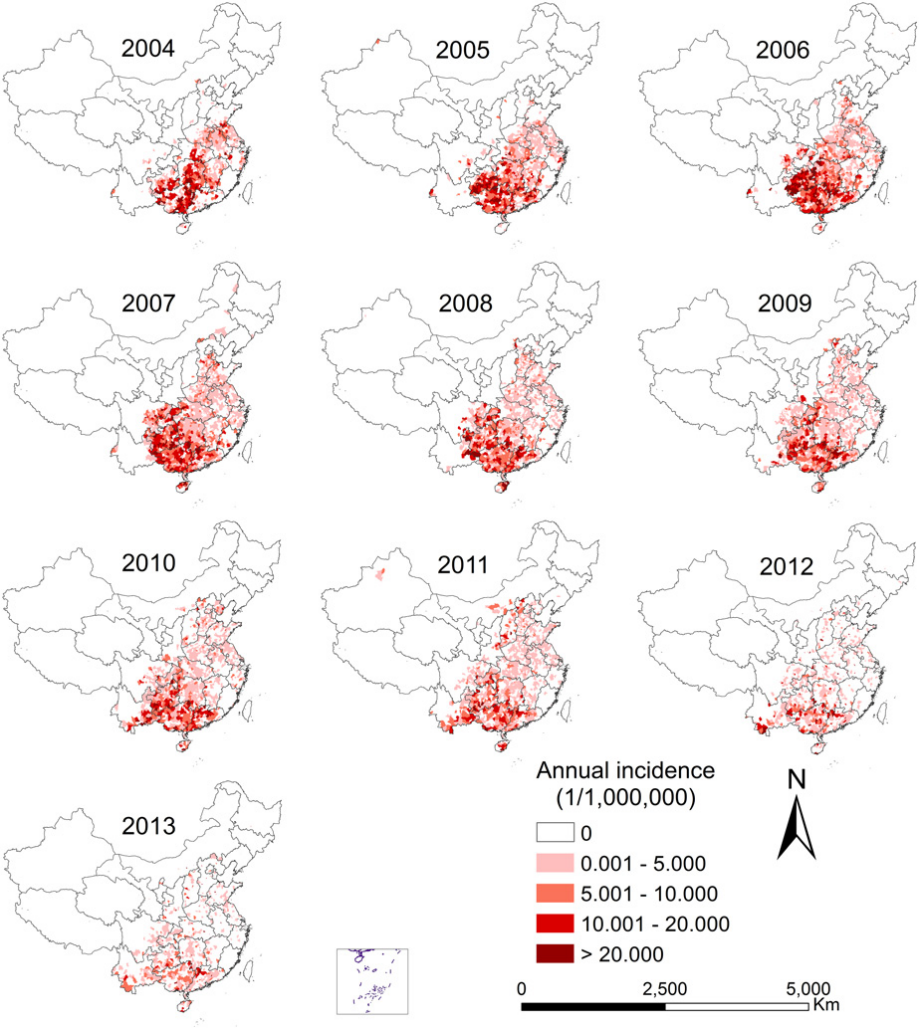
Spatiotemporal distribution of human rabies incidence in mainland China, 2004–2013.

**Fig 4:**
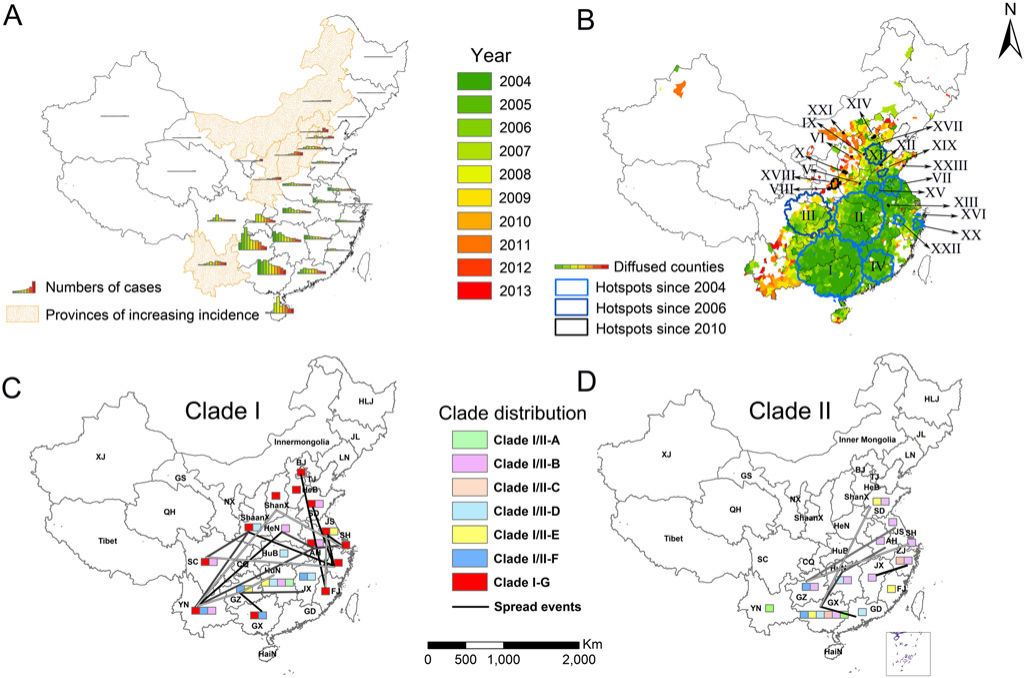
The spatiotemporal dynamic and phylodynamic of Rabies in mainland China. (A) The number of cases at province level over years and provinces of increasing incidence from 2004 to 2013 (B) The diffused counties distribution after 2004 and hotspots movement of human rabies at county level over years (from HS-I to HS-XXIII). The distribution of different sub-clades in clade I (C), clade II (D) and their inferred spread events reconstructed from their MCC trees. The darker line indicates the stronger relative support. Provinces in the figure were coded as follows: HLJ-Heilongjiang, JL-Jilin, LN-Liaoning, BJ-Beijing, TJ-Tianjin, HeB-Hebei, HeN-Henan, ShanX-Shanxi, ShaanX-Shaanxi, SD-Shandong, JS-Jiangsu, SH-Shanghai, AH-Anhui, ZJ-Zhejiang, HuB-Hubei, HuN-Hunan, JX-Jiangxi, FJ-Fujian, GD-Guangdong, GX-Guangxi, YN-Yunnan, HaiN-Hainan, GZ-Guizhou, CQ-Chongqing, NX-Ningxia, SC-Sichuan, QH-Qinghai, GS-Gansu, XJ-Xinjiang, TW-Taiwan.

During 2004–2013, 30 of the 31 provinces in China (except Tibet) reported human rabies cases, and the high-incidence provinces were mostly in the southern, the eastern and part of the central China. The high-incidence provinces were mainly in the south, such as Guizhou, Guangxi, Guangdong and Hunan provinces (Fig. 3). Overall, a decreasing annual incidence was found in the high-incidence provinces, while the low-incidence provinces had an increasing incidence (i.e. Shaanxi, Shanxi, and Yunnan) over the 10 years. This phenomenon is more clearly shown by the mapped number of cases across years at the provincial level and provinces with an increasing pattern over the years were shaded by orange dots (Fig. 4A).

### Spatiotemporal distribution and movement of hotspots

As shown in Fig. 4B, the endemic areas were expanding from areas with green color grads to red color grads over the 10 years, and the hotspots were moving towards the west and the north over the years. The primary hotspot of human rabies (HS-I) was located in southern China and included 281 counties, covering most of Guangxi, Hunan, and Guizhou provinces and spanning from January of 2004 to November of 2012. The relative risk (RR) of reporting human rabies, as compared to the reference regions, was 6.65 for the primary hotspot (Table 1). The hotspots were mainly distributed in the southern, southeastern and central regions before 2006 (HS-I, HS-II, HS-IV, HS-VII, HS-XV, HS-VI HS-XX and HS-XXII), and shifted towards the north (HS-XI) and the west (HS-III) during 2006–2008, and further towards the northwest after 2010 (HS-V, HS-VI, HS-X, HS-XIV, HS-XVII and HS-XXI). Many latest hotspots identified during 2012 to 2013, most of which contained only one county with a high RR (e.g. HS-V, HS-VI), were located in the north and the west, and had very few human cases before 2009.

**Table 1:** Spatiotemporal hotspots of rabies defined by using Spatiotemporal scan statistic, mainland China.*

Hotspots	Start time	End time	No. Couties	No. Obs	No. Exp	LLR	RR
HS-I	2004/1/1	2012/11/30	281	8769	1966.48	7573.78	6.65
HS-II	2004/5/1	2007/11/30	217	1577	776.13	332.00	2.11
HS-III	2006/8/1	2008/10/31	146	872	355.31	272.23	2.51
HS-IV	2004/3/1	2010/12/31	61	796	340.36	225.32	2.39
HS-V	2012/1/1	2013/11/30	1	79	1.86	219.26	42.68
HS-VI	2012/1/1	2013/12/31	1	53	1.73	130.31	30.79
HS-VII	2004/1/1	2005/1/31	40	186	59.46	85.94	3.15
HS-VIII	2012/1/1	2013/8/31	5	72	9.65	82.45	7.48
HS-IX	2012/1/1	2013/11/30	1	32	1.52	66.99	21.04
HS-X	2010/10/1	2013/12/31	1	45	4.42	63.91	10.21
HS-XI	2006/7/1	2007/11/30	66	184	72.91	59.51	2.54
HS-XII	2012/2/1	2013/12/31	1	29	1.99	50.70	14.59
HS-XIII	2012/6/1	2013/12/31	1	25	2.21	37.91	11.34
HS-XIV	2012/1/1	2013/10/31	6	71	22.23	33.73	3.20
HS-XV	2004/5/1	2004/8/31	23	52	13.57	31.47	3.84
HS-XVI	2004/6/1	2005/1/31	1	19	1.60	29.62	11.89
HS-XVII	2012/2/1	2013/12/31	1	25	3.21	29.57	7.81
HS-XVIII	2011/6/1	2011/12/31	9	28	4.65	26.94	6.03
HS-XIX	2006/6/1	2006/11/30	9	32	6.17	26.87	5.20
HS-XX	2004/8/1	2006/12/31	8	54	17.70	23.96	3.06
HS-XXI	2012/2/1	2013/9/30	1	12	0.79	21.50	15.28
HS-XXII	2004/1/1	2004/2/29	8	10	0.53	19.94	18.94
HS-XXIII	2012/5/1	2013/10/31	2	18	2.58	19.57	6.99

*Significant clusters with P<0.05;

HS-I: Primary hotspot;

HS-II—HS-XXIII: Secondary hotspots;

No. Counties: number of counties within hotspots; No. Obs: number of observed cases; No. Exp: number of expected cases; LLR: log likelihood ratio; RR: relative risk for the hotspot compared with the rest of the country.

### Risk factors associated with the incidence of human rabies

The univariate analyses revealed that the spatial-temporal distribution of rabies incidence was associated with monthly temperature at time-lags from 1 to 2 months, GDP per capita, and average education years (Table 2). The multivariate analysis showed that the disease incidence was positive correlation with temperature at a one-month lag (PC = 19.1%; 95% CI = 18.0%, 20.3%), and negative correlation with GDP per capita (PC = -5.7%, 95% CI = -10.2%, -1.0%) and average education years (PC = -13.7%; 95% CI = -17.6%, -9.7%). The risk ratios were similar to those obtained from the univariate analyses, except that the effect size of GDP per capita is smaller. In addition, we found that monthly incidence of human rabies was highly correlated with monthly number of canine rabies outbreaks at a 2-month lag (PC = 11.6%; 95% CI = 10.6%, 12.6%) and temperature at a one-month lag (PC = 3.0%; 95% CI = 2.7%, 3.2%), and each additional canine rabies outbreak will increase the risk of human rabies by about 11.6%.

**Table 2:** The association between human rabies incidence and relevant factors by panel Poisson regression.

Variables (unit)	Univariate analysis		Multivariate analysis	
	Crude PC (95% CI)	P-value	Adjusted PC (95% CI)	P-value
Temperature(5°C)				
Lag0	13.7 (12.7,14.7)	<0.001		
Lag1	19.0 (17.8,20.2)	<0.001	19.1 (18.0,20.3)	<0.001
Lag2	15.2 (14.0,16.3)	<0.001		
GDP per capita(1000 yuan)	-11.8 (-16.0, -7.3)	<0.001	-5.7 (-10.2, -1.0)	0.019
Education year(1 year)	-15.3 (-19.0, -11.5)	<0.001	-13.7 (-17.6, -9)	<0.001

### Phylogenetic tree construction and phylodynamic inference

**Fig 5:**
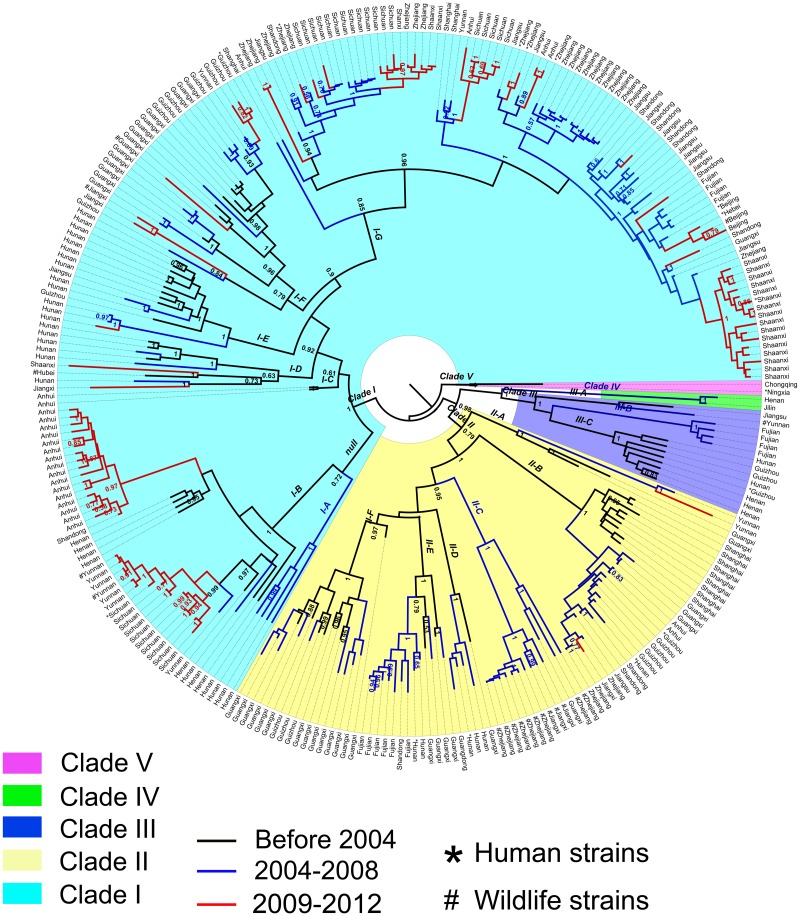
Maximum Clade Credibility tree of N Genes of Rabies in mainland China.

There were a total of five lineages with high posterior node probabilities (>0.5), while only two main lineages, Clade I and Clade II, contributed to rabies epidemic in mainland China from 2004 to 2013 (Fig. 5). In addition, seven sub-clades with high posterior value were identified in Clade I, and six in Clade II. Interestingly, the sequences from 2009 to 2012 were mostly clustered in Clade I (colored red in Fig. 5), indicating the dominant role of Clade I in recent rabies epidemics. The genetic diversities of Clade I and Clade II were mapped at the provincial level in Fig. 4C and 4D. The southern and southwestern provinces, in particular Hunan and Yunnan, had more genetic diversities than the northern and eastern provinces, such as Shanxi, Hebei, Beijing, Shanghai, Zhejiang and Fujian, which were mostly associated with Clade I-G. The abundant genetic diversity of rabies in Guangxi and Hunan province (Fig. 4C and 4D) suggested they might be the center for rabies circulation and evolution. Fig. 5 and 4C imply possible diffusion of Clade I-G from eastern region (e.g., Shanghai, Zhejiang) to southwestern (Sichuan) and northern (Shaanxi) regions and of Clade I-B from Henan to Anhui and Yunnan in recent years. It remains to be verified whether these two clades have gained enhanced fitness or transmissibility.

To further explore the diffusion pattern implied by genetic linkage while accounting for phylogenetic uncertainty, we summarized rates yielding a Bayes factor >3 in Figs. 4C and 4D and S3 Table, which reveal more spread events of Clade I than that of Clade II. Interestingly, in Clade I, we found some rabies spread events that could be related to the spread of human rabies from the south towards the north and west. The migrations of Clade I-G between Shaanxi and Zhejiang and between Shaanxi and Yunnan were strongly supported with high Bayes factors 33.97 and 12.36. Shanxi and Shaanxi, which have latest hotspots of human rabies, were involved in two and three migration pairs with Bayes factor >3 for Clade I-G (Fig. 4B). Additionally, spread events could have also occurred between southwestern provinces (Sichuan, Yunnan) and central, eastern and southern provinces (Henan, Anhui, Zhejiang and Guizhou).

## Discussion

Rabies is considered one of the most dangerous but neglected diseases in developing countries, with the greatest burden in the poorest rural communities where 15 million people need PEP every year [28]. In China, rabies is thought to be under-reported or under-recognized, resulting in an under-estimation of the true disease burden [7].The State Council issued official notices in 2009 and 2012 underlining rabies control as a priority with control objectives between 2015 and 2020 [29,30]. Our study highlighted both the strengths and gaps in the rabies control efforts in China. Despite the decline in human rabies cases at the national level since 2008, especially successful control in historically high-incidence provinces, China has to face a new complication that rabies started to spread from high-incidence regions towards low-incidence regions, as evidenced in our statistical and phylogeographic analyses.

Our findings reveal that the higher temperature, the higher risk of human rabies, which is consistent with the observed peak season in Summer and Autumn. Such seasonality is not surprising, as people tend to wear less and have more outdoor activities (in the absence of indoor air-conditioning in rural areas) in warm and hot weather, and hence have more frequent contact with canines, irascibility of which is sensitive to high temperatures. It also seems reasonable that the male incidence is far higher than the female incidence, as in rural China men tend to have more outdoor activities than women due to cultural factors [31]. The newly-established endemic counties or hotspots mostly occurred in the west or north to the previous endemic areas and represented a trend of spreading of the virus from the southern and eastern regions to the northern and western regions (Fig. 3, 4). Such movement might be explained by the fact that interventions were implemented in baseline high-incidence regions over the years [32] whereas the baseline low-incidence regions started to suffer the increasing burden of rabies in the absence of interventions.

Our analyses revealed the importance of improving the education level and economic status in reducing human rabies incidence in mainland China, and partially explained why farmers, students, and pre-school children are the high risk groups [11]. It is believed that the unawareness of rabies risk and the high price of the rabies PEP are the main reasons for the high incidence in the population with low education and low income [8].The decreasing incidence in rural areas of high-incidence provinces might be partially due to recent efforts in educational campaign about rabies and the introduction of new rural cooperative medical subsidies for PEP costs [32]. In addition, the health services and medical standards for rabies diagnosis and treatment are believed to be other key factors affecting human rabies incidence. For example, the country’s investment in training health professionals on PEP and increasing access to PEP in the countryside likely contributed to the decrease in human rabies cases after 2007 [7,32]. In this study, however, we were not able to quantify the contribution of any intervention due to the lack of data. However, we urge relevant agencies and organizations to extend these intervention programs to rural areas with historical low incidences, in particular around the hotspots we identified.

The rapid economic development in China has greatly encouraged the ownership and transportation of domestic dogs, yet the vaccination of dogs is left far behind [11,32], facilitating the rabies spread. With the help of phylogeographic analysis, some long-distance spread events were detected and support the geographic dispersion pattern found in our epidemiological investigation. Clade I of rabies, especially Clade I-G, was found to dominate recent spread across provinces. These results suggest that the movement of hotspots of human rabies may be a consequence of long distance instead of cross-border migration of hosts. However, interpretation of these results should be cautious as potential sampling bias of sequences could distort the underlying truth. Additionally, although having contributed to most rabies epidemics in the past decade, Clade II was playing a less significant role in the epidemic since 2009. The emergence of the Clade I and the gradual displacement of Clade II are also obvious in recent studies [3,6,33,34]. Our phylodynamic analyses highlight the importance of closely monitoring Clade I in the future and the need to strengthen canine rabies surveillance, to regulate interprovincial animal trade, and to intervene promptly upon detection of canine rabies outbreaks. The usefulness of efficient surveillance on canine rabies is also evidenced by our identification of the positive association of human rabies outbreaks with the canine counterparts at a two-month lag.

While some developed countries, such as the United States and Western European countries had achieved their goal of eliminating human rabies cases, most developing countries, especially those in Africa and Asia, continue to suffer from the burden of rabies and account for 95% of global human cases [35,36]. The magnitudes and epidemiological patterns of rabies differ from country to country [37]. Taking countries bordering China for example, India has the highest rate of human rabies in the world, and its rabies incidence has been nearly constant for a decade. Thailand is moving towards low endemic status, and Indonesia sees increase in incidence and expansion in range. Despite these differences, these countries share some common risk factors, e.g., educational level, economic status and canine rabies [35,36]. A certain level of internationally coordinated efforts such as communication about successful control experiences may be helpful towards global elimination of human rabies [37].

Our study shares some similar limitations with previous studies. The data were collected from a passive surveillance system, which may underreport cases of human rabies especially in rural and remote areas. If underreporting is truly more severe in rural and remote areas where socioeconomic and educational levels are usually lower, the magnitude of the impact of GDP and education levels might have been underestimated. However, this underreporting should have small influence on our results for clustering and expansion of the epidemics, as the hotspots identified in this study are of large scale and encompass both developed and underdeveloped areas. The lack of some highly relevant data such as dog density and dog vaccine coverage also limited our understanding of the true effects of the available risk factors and the driving reason for the declining trend of the overall epidemic. Additionally, GDP per capita and average education years used in our panel Poisson regression were from a single year instead of all the study years. Nevertheless, these data were usually relatively stable over a few years, and spatial heterogeneity generally dominates over temporal changes. Moreover, Bayesian CAR could be used in the panel Poisson model to account for spatial and temporal correlations due to similarity in unmeasured risk factors, which is subject to future research [38]. Finally, despite the large geographic coverage of the sequences used in our phylogeographic analysis, the lack of a sufficient number of sequences at some high-incidence locations such as Guangdong may have led to an incomplete picture of the genotypic distribution and spread of rabies in mainland China.

Overall, we outlined a clear picture of the epidemic patterns of human rabies in China using a multi-disciplinary spatiotemporal analytical approach, and this approach is an integral part in the design of effective elimination strategies for human rabies [35]. We reiterate that control efforts should focus on not only the high-incidence areas but also the low-incidence and emerging areas in order to achieve the goal of rabies elimination by 2020 [13]. The enhanced surveillance of human rabies cases, effective intervention programs, and efficient cooperation among relevant agencies have worked together to achieve the success in rabies control in high-incidence areas since 2007 [32,39,40]. Elimination of rabies is feasible and can be cost effective [41,42]. However, the new challenge of geographic expansion of the rabies epidemic needs to be addressed together with traditional challenges, e.g., the canine immunization coverage may be far below 70%, a critical threshold for interrupting rabies virus circulation in the dog population [1]. We recommend supplementing current control strategies with the allocation of appropriate amount of intervention resources to low-incidence areas, especially areas with limited access to educational and economic development.

### Conclusions

The reduction of human case reports has been observed in mainland China but was accompanied with geographic expansion towards the north and the west. Our multidisciplinary study identified this new challenge with both epidemiological and phylogenetic evidence, and provided new insights on risk factors and control strategies for the disease spread.

## Supporting Information

S1 Table The strains’ information used in this study. (DOCX)

S2 Table Parameter settings for phylodynamic analysis. (DOCX)

S3 Table Bayes factors value between two provinces. (DOCX)

S1 Checklist STROBE Checklist. (DOC)
