# Supplementary material for: The Spatiotemporal Expansion of Human Rabies and Its Probable Explanation in Mainland China, 2004-2013
Source: PLoS Negl Trop Dis. 2015 Feb 18;9(2):e0003502. doi: 10.1371/journal.pntd.0003502 (PMC4334667; doi:10.1371/journal.pntd.0003502)
Supplement: S1 Table — (DOCX) [file pntd.0003502.s001.docx]

**Table S1.** The strains’ information used in this study.

| **Locus** | **Length** | **Collection_date** | **Host** | **Location** | **Clade** | **Discrete analysis** |
| --- | --- | --- | --- | --- | --- | --- |
| DQ666287 | 1484 | 2004 | canine | Guangxi | I | √ |
| DQ666291 | 1484 | 2004 | canine | Guizhou | I | √ |
| DQ666294 | 1484 | 2004 | canine | Guizhou | I |  |
| DQ666295 | 1484 | 2004 | canine | Guizhou | I |  |
| DQ666296 | 1484 | 2004 | canine | Guizhou | I | √ |
| DQ666297 | 1484 | 2004 | canine | Henan | I | √ |
| DQ666298 | 1484 | 2004 | canine | Henan | I | √ |
| DQ666299 | 1484 | 2004 | canine | Henan | I | √ |
| DQ666300 | 1484 | 2004 | canine | Henan | I |  |
| DQ666302 | 1484 | 2004 | canine | Henan | I |  |
| DQ666304 | 1484 | 2004 | canine | Henan | I |  |
| DQ666306 | 1484 | 2004 | canine | Henan | I | √ |
| DQ666307 | 1484 | 2004 | canine | Hunan | I | √ |
| DQ666308 | 1484 | 2004 | canine | Hunan | I | √ |
| DQ666309 | 1484 | 2004 | canine | Hunan | I | √ |
| DQ666311 | 1484 | 2004 | canine | Hunan | I |  |
| DQ666312 | 1484 | 2004 | canine | Hunan | I |  |
| DQ666314 | 1484 | 2004 | canine | Hunan | I |  |
| DQ666315 | 1484 | 2004 | canine | Hunan | I |  |
| DQ666316 | 1484 | 2004 | canine | Hunan | I | √ |
| DQ666317 | 1484 | 2004 | canine | Hunan | I | √ |
| DQ666318 | 1484 | 2004 | canine | Hunan | I | √ |
| DQ666319 | 1484 | 2004 | canine | Hunan | I | √ |
| DQ666321 | 1484 | 2004 | canine | Jiangsu | I | √ |
| DQ866107 | 1353 | 2003 | canine | Guangxi | I | √ |
| DQ866113 | 1353 | 2003 | canine | Guangxi | I | √ |
| DQ866115 | 1353 | 2003 | canine | Guangxi | I | √ |
| DQ866117 | 1353 | 2004 | canine | Guangxi | I | √ |
| DQ866118 | 1353 | 2005 | canine | Guangxi | I | √ |
| EF611081 | 1425 | 2004 | Buffalo | Hubei | I | √ |
| EU008919 | 1353 | 2005 | canine | Hunan | I | √ |
| EU008920 | 1353 | 2005 | canine | Hunan | I |  |
| EU008921 | 1353 | 2005 | canine | Hunan | I |  |
| EU008922 | 1353 | 2005 | canine | Hunan | I |  |
| EU008923 | 1353 | 2005 | canine | Hunan | I | √ |
| EU095330 | 1353 | 2006 | canine | Yunnan | I | √ |
| EU275244 | 1353 | 2007 | canine | Yunnan | I | √ |
| EU275245 | 1353 | 2007 | canine | Yunnan | I | √ |
| EU700031 | 1353 | 2007 | Homo sapiens | Beijing | I | √ |
| EU700032 | 1353 | 2008 | Homo sapiens | Zhejiang | I | √ |
| FJ712193 | 11925 | 2008 | canine | Zhejiang | I | √ |
| FJ712194 | 11925 | 2008 | canine | Zhejiang | I | √ |
| FJ719760 | 1353 | 2008 | canine | Zhejiang | I | √ |
| FJ866827 | 1353 | 2008 | canine | Fujian | I | √ |
| FJ866828 | 1353 | 2008 | canine | Fujian | I |  |
| FJ866835 | 11924 | 2008 | canine | Fujian | I |  |
| FJ866836 | 11924 | 2008 | canine | Fujian | I | √ |
| GQ472469 | 1353 | 2007 | canine | GX-Longan | I | √ |
| GQ472471 | 1353 | 2007 | canine | GX-Liucheng | I | √ |
| GQ472472 | 1353 | 2007 | canine | GX-Nanning | I | √ |
| GQ472474 | 1353 | 2007 | canine | GX-Panlong | I | √ |
| GQ472475 | 1353 | 2005 | canine | GX-Pinxiang | I | √ |
| GQ472476 | 1353 | 2006 | canine | GX-Pinxiang | I | √ |
| GQ472478 | 1353 | 2007 | canine | GX-Yizhou | I | √ |
| GU345748 | 11924 | 2006 | canine | Shanghai | I | √ |
| GU591790 | 1353 | 2009 | canine | Shaanxi | I |  |
| GU591792 | 1353 | 2009 | canine | Sichuan | I | √ |
| GU994209 | 1353 | 2009 | Homo sapiens | Zhejiang | I | √ |
| HM486349 | 1353 | 2006 | canine | Jiangsu | I | √ |
| HM486350 | 1353 | 2006 | canine | Jiangsu | I | √ |
| HM486351 | 1353 | 2006 | canine | Jiangsu | I | √ |
| HM486352 | 1353 | 2006 | canine | Jiangsu | I | √ |
| HM486353 | 1353 | 2005 | canine | Jiangsu | I | √ |
| HM486354 | 1353 | 2006 | canine | Jiangsu | I | √ |
| HM486360 | 1353 | 2005 | canine | Anhui | I | √ |
| HM486363 | 1353 | 2006 | canine | Jiangsu | I | √ |
| HM486376 | 1353 | 2008 | canine | Shandong | I | √ |
| HM486377 | 1353 | 2008 | canine | Shandong | I | √ |
| HM486378 | 1353 | 2007 | canine | Shandong | I | √ |
| HM756692 | 1353 | 2008 | canine | Hunan | I |  |
| HQ118101 | 1353 | 1989 | canine | Anhui | I | √ |
| HQ118102 | 1353 | 2006 | canine | Guizhou | I | √ |
| HQ118103 | 1353 | 2008 | canine | Zhejiang | I | √ |
| HQ118104 | 1353 | 1989 | canine | Anhui | I | √ |
| HQ118105 | 1353 | 2008 | canine | Zhejiang | I |  |
| HQ118106 | 1353 | 2008 | canine | Zhejiang | I | √ |
| HQ118107 | 1353 | 2008 | canine | Zhejiang | I | √ |
| HQ118108 | 1353 | 2008 | canine | Zhejiang | I | √ |
| HQ118109 | 1353 | 2008 | canine | Zhejiang | I | √ |
| HQ118110 | 1353 | 2008 | canine | Zhejiang | I |  |
| HQ118111 | 1353 | 2008 | canine | Zhejiang | I |  |
| HQ695732 | 1353 | 2009 | canine | Shaanxi | I | √ |
| JN974826 | 1353 | 2006 | canine | Guangxi | I | √ |
| JN974827 | 1353 | 2006 | canine | Guangxi | I | √ |
| JN974830 | 1353 | 2005 | canine | Guizhou | I | √ |
| JN974832 | 1353 | 2009 | Homo sapiens | Guizhou | I | √ |
| JN974833 | 1353 | 2010 | canine | Guizhou | I | √ |
| JN974834 | 1353 | 2010 | canine | Guizhou | I | √ |
| JN974835 | 1353 | 2010 | Homo sapiens | Hebei | I | √ |
| JN974836 | 1353 | 2005 | canine | Hunan | I | √ |
| JN974837 | 1353 | 2005 | canine | Hunan | I | √ |
| JN974839 | 1353 | 2005 | canine | Hunan | I | √ |
| JN974840 | 1353 | 2006 | canine | Hunan | I | √ |
| JN974844 | 1353 | 2007 | canine | Hunan | I | √ |
| JN974846 | 1353 | 2008 | canine | Hunan | I | √ |
| JN974848 | 1353 | 2009 | canine | Hunan | I | √ |
| JN974849 | 1353 | 2008 | Homo sapiens | Jiangsu | I | √ |
| JN974850 | 1353 | 2008 | canine | Jiangsu | I | √ |
| JN974851 | 1353 | 2008 | canine | Jiangsu | I | √ |
| JN974852 | 1353 | 2008 | canine | Jiangsu | I | √ |
| JN974853 | 1353 | 2009 | canine | Jiangxi | I | √ |
| JN974855 | 1353 | 2009 | canine | Jiangxi | I | √ |
| JN974856 | 1353 | 2008 | canine | Sichuan | I | √ |
| JN974857 | 1353 | 2008 | canine | Sichuan | I | √ |
| JN974858 | 1353 | 2009 | canine | Sichuan | I | √ |
| JN974859 | 1353 | 2009 | canine | Sichuan | I |  |
| JN974860 | 1353 | 2009 | canine | Sichuan | I | √ |
| JN974861 | 1353 | 2010 | canine | Sichuan | I | √ |
| JN974862 | 1353 | 2010 | canine | Sichuan | I | √ |
| JN974863 | 1353 | 2010 | canine | Sichuan | I | √ |
| JN974864 | 1353 | 2008 | canine | Shandong | I | √ |
| JN974865 | 1353 | 2008 | canine | Shandong | I | √ |
| JN974866 | 1353 | 2009 | canine | Shandong | I | √ |
| JN974867 | 1353 | 2009 | canine | Shandong | I | √ |
| JN974868 | 1353 | 2009 | canine | Shandong | I | √ |
| JN974869 | 1353 | 2009 | canine | Shandong | I | √ |
| JN974874 | 1353 | 2005 | canine | Shanghai | I | √ |
| JN974875 | 1353 | 2005 | canine | Shanghai | I | √ |
| JN974876 | 1353 | 2009 | canine | Shanxi | I | √ |
| JN974878 | 1353 | 2008 | canine | Zhejiang | I | √ |
| JQ423952 | 11924 | 2011 | equine | Beijing | I | √ |
| JQ794536 | 1353 | 2011 | donkey | Yunnan | I |  |
| JQ794537 | 1353 | 2011 | canine | Yunnan | I |  |
| JQ794538 | 1353 | 2011 | canine | Yunnan | I | √ |
| JQ794539 | 1353 | 2011 | canine | Yunnan | I |  |
| JQ794540 | 1353 | 2011 | mule | Yunnan | I |  |
| JQ794541 | 1353 | 2011 | canine | Yunnan | I |  |
| JQ798943 | 1353 | 2011 | canine | Anhui | I |  |
| JQ798944 | 1353 | 2011 | canine | Anhui | I |  |
| JQ798945 | 1353 | 2011 | canine | Anhui | I | √ |
| JQ798946 | 1353 | 2011 | canine | Anhui | I | √ |
| JQ798947 | 1353 | 2011 | canine | Anhui | I |  |
| JQ798948 | 1353 | 2011 | canine | Anhui | I |  |
| JQ798949 | 1353 | 2011 | canine | Anhui | I |  |
| JQ798950 | 1353 | 2011 | canine | Anhui | I |  |
| JQ798951 | 1353 | 2011 | canine | Anhui | I |  |
| JQ798952 | 1353 | 2011 | canine | Anhui | I |  |
| JQ798953 | 1353 | 2011 | canine | Anhui | I |  |
| JQ798954 | 1353 | 2011 | canine | Anhui | I | √ |
| JQ798955 | 1353 | 2011 | canine | Anhui | I | √ |
| JQ798956 | 1353 | 2011 | canine | Anhui | I | √ |
| JQ798957 | 1353 | 2011 | canine | Anhui | I |  |
| JQ798958 | 1353 | 2011 | canine | Anhui | I |  |
| JQ798959 | 1353 | 2011 | canine | Anhui | I |  |
| JQ798960 | 1353 | 2011 | canine | Anhui | I | √ |
| JQ798961 | 1353 | 2011 | canine | Anhui | I |  |
| JX005929 | 1353 | 2008 | canine | Sichuan | I | √ |
| JX005930 | 1353 | 2010 | canine | Sichuan | I | √ |
| JX005931 | 1353 | 2008 | canine | Sichuan | I |  |
| JX005932 | 1353 | 2008 | canine | Sichuan | I |  |
| JX005933 | 1353 | 2008 | canine | Sichuan | I |  |
| JX005934 | 1353 | 2008 | canine | Sichuan | I | √ |
| JX005935 | 1353 | 2009 | canine | Sichuan | I | √ |
| JX005936 | 1353 | 2010 | canine | Sichuan | I | √ |
| JX005937 | 1353 | 2008 | canine | Sichuan | I |  |
| JX005938 | 1353 | 2008 | canine | Sichuan | I | √ |
| JX005939 | 1353 | 2008 | canine | Sichuan | I |  |
| JX005940 | 1353 | 2009 | canine | Sichuan | I |  |
| JX005941 | 1353 | 2009 | canine | Sichuan | I | √ |
| JX005942 | 1353 | 2009 | canine | Sichuan | I | √ |
| JX005943 | 1353 | 2010 | Homo sapiens | Sichuan | I | √ |
| JX005944 | 1353 | 2009 | canine | Sichuan | I | √ |
| JX005945 | 1353 | 2010 | canine | Sichuan | I | √ |
| JX005946 | 1353 | 2010 | canine | Sichuan | I |  |
| JX005947 | 1353 | 2010 | canine | Sichuan | I | √ |
| JX123685 | 1353 | 2010 | canine | Zhejiang | I |  |
| JX123686 | 1353 | 2010 | canine | Zhejiang | I | √ |
| JX123687 | 1353 | 2010 | Homo sapiens | Zhejiang | I | √ |
| JX123688 | 1353 | 2009 | Homo sapiens | Zhejiang | I | √ |
| KC169986 | 11923 | 2009 | rabid cattle | GX-Hengxian, | I | √ |
| KC660078 | 11815 | 2012 | canine | BJ-Fengtai | I | √ |
| KC762941 | 11923 | 2009 | Melogale moschata | Jiangxi | I | √ |
| KF663511 | 1353 | 2011 | canine | SX-Xi'An | I | √ |
| KF663512 | 1353 | 2011 | canine | SX-Xi'An | I |  |
| KF663513 | 1353 | 2011 | canine | SX-Xi'An | I | √ |
| KF663514 | 1353 | 2011 | canine | SX-Xi'An | I | √ |
| KF663515 | 1353 | 2011 | canine | SX-Xi'An | I |  |
| KF663516 | 1353 | 2011 | canine | SX-Xi'An | I | √ |
| KF663517 | 1353 | 2011 | canine | SX-Xi'An | I |  |
| KF663518 | 1353 | 2011 | canine | SX-Xi'An | I | √ |
| KF663519 | 1353 | 2011 | canine | SX-Xi'An | I |  |
| KF663520 | 1353 | 2011 | canine | SX-Xi'An | I | √ |
| KF663521 | 1353 | 2012 | canine | SX-Weinan | I | √ |
| KF663522 | 1353 | 2012 | canine | SX-Xianyang | I | √ |
| KF663523 | 1353 | 2009 | canine | ZJ-Hangzhou | I | √ |
| KF663524 | 1353 | 2010 | canine | ZJ-Hangzhou | I | √ |
| KF663525 | 1353 | 2009 | canine | ZJ-Hangzhou | I | √ |
| KF663526 | 1353 | 2011 | canine | SX-Xi'An | I |  |
| KF663527 | 1353 | 2012 | canine | SX-Xi'An | I | √ |
| KF663528 | 1353 | 2012 | Homo sapiens | SX-Xi'An | I | √ |
| KF663529 | 1353 | 2012 | canine | SX-Weinan | I | √ |
| KF663530 | 1353 | 2012 | canine | SX-Xianyang | I | √ |
| DQ666289 | 1484 | 2004 | canine | Guizhou | II | √ |
| DQ666290 | 1484 | 2004 | canine | Guizhou | II | √ |
| DQ866105 | 1353 | 2004 | canine | Guangxi | II | √ |
| DQ866106 | 1353 | 2003 | canine | Guangxi | II | √ |
| DQ866108 | 1353 | 2003 | canine | Guangxi | II | √ |
| DQ866109 | 1353 | 2003 | canine | Guangxi | II | √ |
| DQ866110 | 1353 | 2004 | canine | Guangxi | II | √ |
| DQ866111 | 1353 | 2000 | canine | Guangxi | II | √ |
| DQ866112 | 1353 | 2004 | canine | Guangxi | II |  |
| DQ866114 | 1353 | 2004 | canine | Guangxi | II | √ |
| DQ866116 | 1353 | 2003 | canine | Guangxi | II | √ |
| DQ866119 | 1353 | 2005 | canine | Guangxi | II | √ |
| DQ866120 | 1353 | 2005 | canine | Guangxi | II | √ |
| DQ866121 | 1353 | 2005 | canine | Guangxi | II | √ |
| EU275243 | 1353 | 2006 | canine | Yunnan | II | √ |
| FJ561726 | 1353 | 2008 | canine | Fujian | II | √ |
| FJ561727 | 1353 | 2008 | canine | Fujian | II | √ |
| FJ561728 | 1353 | 2008 | canine | Fujian | II | √ |
| FJ594278 | 1353 | 1997 | canine | Guangxi | II | √ |
| FJ598135 | 1353 | 2008 | Ferret badger | Zhejiang | II | √ |
| FJ712195 | 11923 | 2008 | Ferret badger | Zhejiang | II | √ |
| FJ712196 | 11923 | 2008 | Ferret badger | Zhejiang | II | √ |
| FJ719751 | 1353 | 2008 | Ferret badger | Jiangxi | II | √ |
| FJ719753 | 1353 | 2008 | Ferret badger | Jiangxi | II | √ |
| FJ719755 | 1353 | 2008 | Ferret badger | Jiangxi | II | √ |
| FJ866829 | 1353 | 2007 | canine | Fujian | II | √ |
| FJ866830 | 1353 | 2007 | canine | Fujian | II | √ |
| FJ866831 | 1353 | 2007 | canine | Fujian | II | √ |
| GQ472468 | 1353 | 2007 | canine | GX-Hengxian | II | √ |
| GQ472470 | 1353 | 2007 | canine | GX-Liubei | II | √ |
| GQ472473 | 1353 | 2007 | canine | GX-Nanning | II | √ |
| GQ472477 | 1353 | 2006 | canine | GX-Qinzhou | II | √ |
| GU358653 | 11922 | 1994 | canine | Guangxi | II | √ |
| HM486355 | 1353 | 2004 | canine | Shanghai | II |  |
| HM486356 | 1353 | 2004 | canine | Shanghai | II |  |
| HM486357 | 1353 | 2003 | canine | Shanghai | II |  |
| HM486358 | 1353 | 2003 | canine | Shanghai | II |  |
| HM486359 | 1353 | 2003 | canine | Shanghai | II |  |
| HM486361 | 1353 | 2005 | canine | Anhui | II |  |
| HM486362 | 1353 | 2005 | canine | Anhui | II | √ |
| HM486364 | 1353 | 2006 | canine | Jiangsu | II | √ |
| HM486365 | 1353 | 2006 | canine | Guangxi | II | √ |
| HM486366 | 1353 | 2005 | canine | Guangxi | II | √ |
| HM486367 | 1353 | 2005 | canine | Guizhou | II | √ |
| HM486368 | 1353 | 2005 | canine | Guizhou | II | √ |
| HM486369 | 1353 | 2006 | canine | Guizhou | II | √ |
| HM486370 | 1353 | 2005 | canine | Guangxi | II |  |
| HM486371 | 1353 | 2005 | canine | Guizhou | II | √ |
| HM486372 | 1353 | 2006 | canine | Guizhou | II | √ |
| HM486373 | 1353 | 2008 | canine | Zhejiang | II | √ |
| HM486374 | 1353 | 2008 | canine | Zhejiang | II |  |
| HM486375 | 1353 | 2008 | canine | Zhejiang | II | √ |
| HM486379 | 1353 | 2007 | canine | Shandong | II | √ |
| HM486380 | 1353 | 2006 | canine | Shandong | II | √ |
| HM486381 | 1353 | 2007 | canine | Shandong | II | √ |
| HQ118114 | 1353 | 2008 | ferret badger | Zhejiang | II |  |
| HQ118115 | 1353 | 2008 | ferret badger | Zhejiang | II |  |
| HQ118116 | 1353 | 2008 | ferret badger | Zhejiang | II |  |
| HQ118117 | 1353 | 2008 | ferret badger | Zhejiang | II |  |
| HQ118118 | 1353 | 2008 | ferret badger | Zhejiang | II |  |
| JN974823 | 1353 | 2008 | canine | Guangdong | II | √ |
| JN974824 | 1353 | 2005 | canine | Guangxi | II | √ |
| JN974825 | 1353 | 2006 | canine | Guangxi | II | √ |
| JN974828 | 1353 | 2006 | canine | Guangxi | II | √ |
| JN974829 | 1353 | 2008 | canine | Guangxi | II | √ |
| JN974831 | 1353 | 2005 | Homo sapiens | Guizhou | II | √ |
| JN974838 | 1353 | 2005 | canine | Hunan | II | √ |
| JN974841 | 1353 | 2006 | Homo sapiens | Hunan | II | √ |
| JN974842 | 1353 | 2006 | canine | Hunan | II | √ |
| JN974843 | 1353 | 2006 | Homo sapiens | Hunan | II | √ |
| JN974845 | 1353 | 2008 | canine | Hunan | II | √ |
| JN974847 | 1353 | 2008 | Homo sapiens | Hunan | II | √ |
| JN974854 | 1353 | 2009 | canine | Jiangxi | II | √ |
| JN974870 | 1353 | 2003 | canine | Shanghai | II |  |
| JN974871 | 1353 | 2003 | canine | Shanghai | II |  |
| JN974872 | 1353 | 2003 | canine | Shanghai | II | √ |
| JN974873 | 1353 | 2004 | canine | Shanghai | II | √ |
| JN974877 | 1353 | 2008 | ferret badger | Zhejiang | II |  |
| JQ730682 | 11923 | 2010 | canine | Yunnan | II | √ |
| DQ666288 | 1484 | 2004 | Homo sapiens | Guizhou | III |  |
| DQ666292 | 1483 | 2004 | canine | Guizhou | III |  |
| DQ666293 | 1484 | 2004 | canine | Guizhou | III |  |
| DQ666301 | 1484 | 2004 | canine | Henan | III |  |
| DQ666303 | 1484 | 2004 | canine | Henan | III |  |
| DQ666305 | 1483 | 2004 | canine | Henan | III |  |
| DQ666310 | 1484 | 2004 | canine | Hunan | III |  |
| DQ666313 | 1483 | 2004 | canine | Hunan | III |  |
| DQ666322 | 1483 | 2004 | canine | Jiangsu | III |  |
| EU282381 | 1353 | 2006 | Mouse | Yunnan | III |  |
| FJ561729 | 1353 | 2008 | canine | Fujian | III |  |
| FJ561730 | 1353 | 2008 | canine | Fujian | III |  |
| FJ561731 | 1353 | 2008 | canine | Fujian | III |  |
| FJ561732 | 1353 | 2008 | canine | Fujian | III |  |
| EU652445 | 1567 | 2007 | Raccoon canine | Jilin | IV |  |
| FJ415313 | 5367 | 2008 | Raccoon canine | Henan | IV |  |
| GU345746 | 11908 | 1992 | canine | Chongqing | V |  |
| GU345747 | 11908 | 1986 | Homo sapiens | Ningxia | V |  |
