# Supplementary material for: The Spatiotemporal Expansion of Human Rabies and Its Probable Explanation in Mainland China, 2004-2013
Source: PLoS Negl Trop Dis. 2015 Feb 18;9(2):e0003502. doi: 10.1371/journal.pntd.0003502 (PMC4334667; doi:10.1371/journal.pntd.0003502)
Supplement: S2 Table — (DOCX) [file pntd.0003502.s002.docx]

**Table S2**.Parameter settings for phylodynamic analysis

| **Sequences** | **best substitution model** | **Length of chain** | **sample frequency** |
| --- | --- | --- | --- |
| All rabies | GTR+G+I^a^ | 50000000 | 1000 |
| China I | GTR+G^b^ | 30000000 | 1000 |
| China II | GTR+G+I | 40000000 | 2000 |

^a^ general time-reversible model of nucleotide substitution with gamma-distributed rate heterogeneity among sites and a proportion of invariant sites.

^b^ general time-reversible model of nucleotide substitution with gamma-distributed rate heterogeneity among sites.
