# Supplementary material for: The Spatiotemporal Expansion of Human Rabies and Its Probable Explanation in Mainland China, 2004-2013
Source: PLoS Negl Trop Dis. 2015 Feb 18;9(2):e0003502. doi: 10.1371/journal.pntd.0003502 (PMC4334667; doi:10.1371/journal.pntd.0003502)
Supplement: S3 Table — (DOCX) [file pntd.0003502.s003.docx]

**Table S3**.Bayes factors value between two provinces.

| **Spread events in lineage China I** | | **Bayes factor** |
| --- | --- | --- |
| Jiangsu | Shanghai | 3168.97 |
| Shaanxi | Zhejiang | 33.97 |
| Guangxi | Guizhou | 29.64 |
| Shangdong | Zhejiang | 27.87 |
| Jiangsu | Zhejiang | 22.96 |
| Beijing | Fujian | 22.39 |
| Shaanxi | Yunnan | 12.36 |
| Auhui | Shanghai | 11.85 |
| Auhui | Yunnan | 7.64 |
| Sichuan | Shaanxi | 6.19 |
| Henan | Yunnan | 6.18 |
| Auhui | Henan | 5.69 |
| Guizhou | Jiangxi | 5.26 |
| Sichuan | Zhejiang | 4.67 |
| Guizhou | Hunan | 4.66 |
| Fujian | Jiangsu | 4.5 |
| Shanghai | Shanxi | 4.03 |
| Guizhou | Yunnan | 3.9 |
| Shanxi | Yunnan | 3.87 |
| **Spread events in lineage China II** | | **Bayes factor** |
| Jiangxi | Zhejiang | 88.1 |
| Guangdong | Guangxi | 40.4 |
| Guangxi | Hunan | 21 |
| Anhui | Hunan | 5.29 |
| Guizhou | Jiangsu | 4 |
| Guangxi | Shangdong | 3.47 |
| Guizhou | Shanghai | 3.29 |
